# Initial Selection of Disc Brake Pads Material based on the Temperature Mode

**DOI:** 10.3390/ma13040822

**Published:** 2020-02-11

**Authors:** Aleksander A. Yevtushenko, Piotr Grzes

**Affiliations:** Faculty of Mechanical Engineering, Bialystok University of Technology (BUT), 45C Wiejska Street, 15-351 Bialystok, Poland; a.yevtushenko@pb.edu.pl

**Keywords:** braking, frictional heating, temperature, wear, material selection, finite element method

## Abstract

A spatial computational model of a motor vehicle disc brake, based on the system of equations of heat dynamics of friction and wear (HDFW), was developed. The interrelations of temperature-dependent coefficient of friction and coefficient of intensity of wear through the contact temperature and vehicle velocity were taken into account. The solution of the system of equations of HDFW was obtained by the finite element method (FEM) for six different brake pad materials associated with the cast-iron disc during a single braking. Changes in the braking time, coefficient of friction, braking torque, vehicle velocity, mean temperature of the contact area of the pads with the disc and wear of the friction surfaces were determined. Then, the obtained calculation results were evaluated in terms of stabilization of the coefficient of friction (braking torque), as well as minimization of the maximum temperature, wear, braking time and pads mass. As a result, recommendations were given to select optimum brake pad material in combination with a cast-iron disc.

## 1. Introduction

The main function of a braking system is to reduce velocity, stopping or preventing movement of a vehicle. Therefore, it is important to obtain sufficiently high and stable braking torque, ensuring stopping of rotating parts (disc and related components). With the given construction dimensions of the brake and the clamping force, a central role plays the coefficient of friction, which is intrinsically connected with the appropriate friction materials selection.

An attempt to systematize the problems of selection of materials for disc brake components was made in article [1]. The object of the analysis were materials used for motor vehicle brake discs. From the group of material selection criteria, the most important were stability (irrespective of load) of coefficients of friction and wear intensity, change in sliding velocity and temperature mode during braking. Among the additional requirements taken into account when selecting friction pair materials, the material properties such as compression strength, density and specific heat capacity were listed. Furthermore, material resistance to cracking, manufacturing and the associated cost should be considered. An important aspect of the material selection process is also the possibility of reprocessing and recycling. The authors drew attention to the problem of the diversity of approaches of material selection methods [2,3,4]. One of the basic ways of selecting friction materials is to develop a diagram (chart) presenting the properties of materials according to the specific type of braking system. Another method is the digital logic (DL) method using Ashby’s chart [5]. The selection of material for the brake disc according to that method proceeds in the following four stages [1]:

(1). general material performance requirements;

The basic requirements for the braking system are taken into account—obtaining the highest possible and stable coefficient of friction. The criteria include compression strength, coefficient of friction, wear resistance, specific heat capacity, material density and costs.

(2). initial screening of the candidate material;

Based on the general requirements, materials are strictly selected for the braking system.

(3). material selection using digital logic method;

First, the weighting factors of the criterion parameters from step (1) should be found. Then, the performance index of each material is calculated. The material with the highest index is considered to be the most useful.

(4). optimum material selection;

The performance index together with the total cost of the material selected in stage (3) is compared with the corresponding parameters of gray cast iron (GCI).

Using the above method, it was found that from the materials considered, namely Ti-alloy (Ti-6Al-4V), 7.5 wt% WC and 7.5 wt% TiC reinforced Ti-composite (TMC), 20% SiC reinforced Al-composite (AMC 1) and 20% SiC reinforced Al-Cu alloy (AMC 2), the highest performance index had AMC 2 material (88.6), and it was larger than the cast iron performance index (81).

The radial cracking process of worn, ventilated automotive brake discs made of gray cast-iron was studied in article [6]. A number of images of the microstructure (crack top view and cross-section) and hardness across the crack at different distances from the contact surface, obtained from optical microscope, scanning electron microscope (SEM), and energy dispersive X-ray spectroscopy (EDS) were shown and discussed. It was stated that the main source of appearance of the straight radial cracks, propagated from the outer edge of the disc, was the excessive wear. Both abrasive and adhesive wear mechanisms were identified in the specimen. Based on the carried out additional pin-on-disc wear tests with gray cast iron, coefficient of friction, weight loss against sliding distance, distribution of the laser profilometry at the end of the process and SEM images of the sample were presented and analyzed.

Thermo-structural analysis for conventional solid and grooved brake discs manufactured based on 3D printed of maraging steel was carried out in article [7]. The numerical calculations of temperature evolutions and von Mises equivalent stress fields in the discs were performed using ANSYS finite element method based software. In addition, the effect of the radial grooves area on the changes in the heat flux and temperature of the brake disc (6, 9 and 18 grooves) was investigated. The existence of grooves cut on the disc surface through Direct Metal Laser Sintering (DMLS) process, led to both lower von Mises stress and lower temperature. That fact was mainly attributed to more efficient heat dissipation.

The conversion of mechanical energy into heat results in an increase in temperature at the interface of two sliding components of the braking system. As established on the basis of a number of experimental studies and calculations, an effect of temperature on tribological characteristics, durability and reliability is undeniable [8]. Due to the dependence of the coefficient of friction on temperature, thermal sensitivity of thermophysical properties of materials, wear and an array of physical phenomena accompanying the friction process, a loop of unstable cause-effect processes takes place. That effect, especially the instability of the coefficient of friction, adversely affects operating conditions and safety. The difficulty in developing universal guidelines in the materials selection stems, among others, from the variety of disc brake designs due to their intended use—there are permissible different contact pressures, velocities, contact surface temperatures and bulk volumetric temperatures achieved, the level of wear and its mechanisms in aircraft brakes, automotive brakes or devices and working machines. Materials are also constantly developed and modified on account of growing requirements for high and stable braking torque while minimizing the mass of the friction pair. One should also remember about the technological features of the material, resistance to weather conditions (susceptibility to oxidation of carbon materials), resistance to various types of liquids (water, oil and other substances), as well as ecological aspects. The requirements for friction materials include stable and high coefficient of friction, low wear irrespective of working conditions, resistance to adhesive tacking, stability and uniformity of changes in chemical and phase composition, other properties of the surface layer during operation, corrosion resistance, high melting point, high thermal conductivity, low thermal expansion coefficient—constant shape and dimensions irrespective of changes in temperature, high specific heat capacity, absence of vibration and squeal noise [9,10].

The integral quantity combining the parameters and factors mentioned above is the temperature of the braking system [11,12]. Its mode (regime) largely determines the friction and wear characteristics of the brake sliding components. Knowing the temperature field, one can perform a preliminary friction pair materials selection. It will answer the next two questions. First, whether the friction material will work at its acceptable temperature, and secondly, what the approximate wear of the working surfaces is, i.e., the service life of the friction pair [13]. To answer that question, it is necessary to have experimental data on the friction stability of the considered pairs of materials—the dependence of their coefficients of friction and the intensity of wear on temperature. They are the basis for the calculation model of the maximum brake temperature using the finite element method and the system of equations of heat dynamics of friction and wear (HDFW) [14,15]. The solution of that system of equations will allow for a comprehensive assessment of the working ability of a preselected friction pair at the stage of developing the brake structure within the foreseeable range of operating parameters. The scheme of friction pair selection based on the solution (analytical, analytical-numerical or numerical) of the HDFW system of equations can be presented as follows [16]:

(1). estimation of the bulk volumetric temperature for the most severe brake operating conditions and selection of the class of materials (polymers, sintered powders, carbon composites, etc.), from which friction materials may be chosen;
(2). calculating the average temperature of the nominal contact area of the friction pair to reduce the number of materials selected and assessing the brake structure for thermal strain and stresses as well as structural changes on the contact surfaces;
(3). determination of the maximum temperature and real changes of the coefficient of friction and wear during braking.

Comparison of different combinations of materials of the friction pair is carried out based on the parameters that largely affect the smoothness of braking. These are [17]:

(1). mean value $f_{m}$ of the coefficient of friction f;
(2). stability $f_{s}=f_{m}/f_{\max}$;
(3). fluctuation $f_{f}=f_{\min}/f_{\max}$;
(4). braking efficiency $\alpha_{eff}=f_{s}/t_{s}^{2}$, where $t_{s}$ is braking time;
(5). relative braking efficiency $\beta_{eff}=\alpha_{eff}/I_{l,\max}$, where $I_{l,\max}$ is maximum value of linear wear $I_{l}$.

These parameters allow the operation of a given pair of materials to be evaluated in terms of meeting all indexes for friction and wear, including the requirement of stability of braking torque and smoothness of the braking process itself. Having such data, the constructor, taking into account the requirements of the machine functioning, can be more justified and more certain in determining the optimum brake option. At the same time, the basic issues of the dilemma should be taken into account: what is more desirable in the case under consideration—braking efficiency or economic aspects, i.e., reducing wear and accordingly increasing the operating time of the brake.

Numerical calculations using the finite element method of the axisymmetric temperature fields of the pads and the disc during a single braking were carried out in the article by Yevtushenko and Grzes [18]. In the braking simulation, 16 configurations of materials of the pad-disc system were examined, which included four materials of the Al MMC (aluminum alloy series), FCD50 (iron alloy series), steel EI-696, cast iron ChNMKh and four materials of pads cermet FMC-11, FMC-845, MCV-50 and titanium alloy VT-14. The main purpose of the study was to carry out comparative analysis of temperature evolutions of the friction surfaces of the brake contact model with the constant and temperature-dependent thermophysical properties of materials. In each of the analyzed cases, the change in velocity was linear (constant deceleration), and thus the total friction power density did not change. Such assumptions allowed direct investigation of the effect of material properties on temperature fields of the brake components. It was found that taking into account the thermally sensitive materials has no significant effect on the maximum temperature values (difference below 3%). Individual pairs revealed larger differences, reaching around 16%. In the analysis of the results obtained, emphasis was placed on thermal effusivity—the parameter being the square root of the product of thermal conductivity, density and specific heat capacity. Its greater change during braking caused the largest differences in surface temperature for constant and thermally sensitive materials.

Axisymmetric (2D) and spatial (3D) computational models of disc brakes using FEM with the temperature-dependent coefficient of friction were developed in articles [19,20]. The temperatures and wear of the friction pairs, including pads made of FC-16L Retinax A or cermet FMC-11, sliding on the surface of the cast-iron disc during single (2D model) and multiple (3D model, 6 brake applications) braking were studied. The same braking time at constant and thermally sensitive coefficient of friction adapted in both models gave different values of the full work done, which made it difficult to estimate the influence of the input parameters on temperature and wear.

On the basis of the 2D numerical solution of the system of equations of HDFW, the influence of disc brake design features on the maximum temperature and duration of the braking process was examined in the article [21]. The dependence of the coefficient of friction on the mean temperature of the contact area of the pad with the disc was taken into account, based on the coupling of the initial value problem for the equation of motion and the boundary value heat conduction problem (thermal problem of friction). That approach allowed the same work done during braking to be performed in each of the analyzed cases (five geometrical variants and four contact pressure values). Five geometrical models differing in the outer diameter of the disc and brake pads were analyzed, while maintaining a constant volume—increasing the diameter led to reduction in the thickness of the brake components. It was established that increasing the equivalent radius of the friction path (outer and inner diameter of the pads and disc) significantly shortens the braking time and distance, while the maximum temperature achieved slight changes. The corresponding 3D thermal problem of friction based on the system of equations of HDFW was studied in article [22]. The calculations were conducted using a 3D contact model of a disc brake. Two friction pairs at six contact pressures, assuming that the properties of materials are constant, were analyzed.

The elastic-plastic effects in the process of Vickers indentation of deep drawing quality steel sheets were investigated using the finite element method in the paper [23]. The authors placed emphasis on correlation of anisotropy of the material according to Hill yield criterion and contact conditions. Nonlinear numerical calculations of stresses and strains were carried out on the basis of the three-dimensional contact model of the 3D rigid indenter and deforming steel sheet with the real thickness. The indenter shape was ideal, without the rounding. In order to assure accuracy of the computer simulation, sensitivity analysis with the different total number of elements of the mesh was performed. Distributions of the equivalent plastic strain along the rolling direction and equivalent stress under maximum displacement and after unloading were presented and analyzed. It was observed that the coefficient of friction affects the hardness of the material, however friction conditions affect the maximum force and the character of the load-displacement correlations slightly. Two-dimensional finite element FE indentation analysis and the experimental Digital Image Correlation (DIC) method were used to study strains for the samples made of a ductile material, 99% tin [24].

The purpose of the research carried out in this work was to develop a methodology for selection of pads material for the optimum friction of a given brake disc taking into account the boundary value heat conduction problem and the initial value problem for the equation of motion. To find the temperature mode of the pad-disc pair, a coupled 3D FEM model was adapted from the article [22]. The calculations were conducted for six brake pad materials associated with the cast-iron disc.

## 2. HDFW System of Equations

The results of experimental tests on the friction thermostability of the materials of the friction pair in question, i.e., changes in the coefficients of friction f and intensity of thermomechanical mass wear I under the influence of temperature T, were approximated by the functions:

(1) $$f(T)=f_{0}f^{*}(T),\;f_{0}\equiv f(T_{0}),I(T)=I_{0}I^{*}(T),\;I_{0}\equiv I(T_{0})$$

(2) $$f^{*}(T)=f_{1}+\frac{f_{2}}{{\left[f_{3}(T-f_{4})\right]}^{2}+1}+\frac{f_{5}}{{\left[f_{6}(T-f_{7})\right]}^{2}+1}$$

(3) $$I^{*}(T)=I_{1}+\frac{I_{2}}{{\left[I_{3}(T-I_{4})\right]}^{2}+1}+\frac{I_{5}}{{\left[I_{6}(T-I_{7})\right]}^{2}+1}$$

where $T_{0}$ is the initial temperature of the system, while the values of the coefficients $f_{i}$, $I_{i}$, i=1, 2,…,7 were found using methodology from [19,25].

It was assumed that the pressure p is uniformly distributed over the nominal contact areas of each of the two brake pads with the single brake disc and increases exponentially in time t from zero to the nominal value $p_{0}$ according to the relationship:

(4) $$p(t)=p_{0}p^{*}(t),\;p^{*}(t)=1-e^{-t/t_{i}},\;0\leq t\leq t_{s},$$

where $t_{i}$ is the rise time and $t_{s}$ is the braking time.

At the initial time moment t=0, the vehicle with mass m, equipped with four wheels with the same dynamic radius $R_{w}$, moves at the initial velocity $V_{0}$. Change in the vehicle velocity V during braking will be found from the solution to the initial value problem for the equation of motion:

(5) $$m\frac{dV(t)}{dt}=-8F(t),\;0<t\leq t_{s},\;V(0)=V_{0},$$

where

(6) $$F(t)=F_{0}F^{*}(t),\;F_{0}=f_{0}p_{0}A_{a}R_{w}^{-1}r_{eq},\;F^{*}(t)=f^{*}[T_{m}(t)]p^{*}(t),$$

(7) $$T_{m}(t)=\frac{1}{A_{a}}\int_{-\theta_{0}}^{\theta_{0}}\int_{r_{p}}^{R_{p}}T(r,\theta ,0,t)rdrd\theta$$

(8) $$r_{eq}=\frac{2(R_{p}^{3}-r_{p}^{3})}{3(R_{p}^{2}-r_{p}^{2})}$$

**Figure 1:**
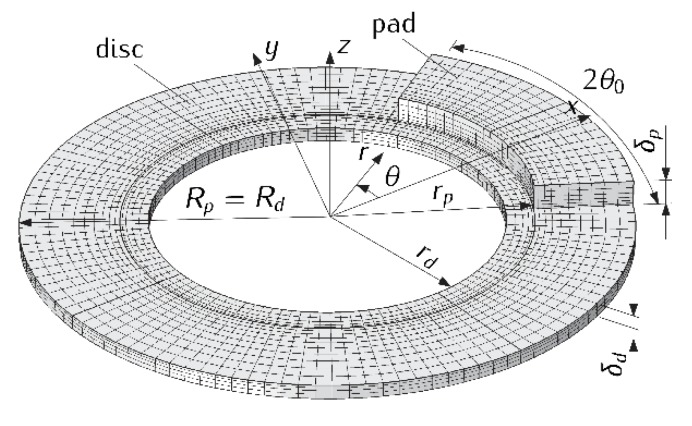
Finite element mesh of the contact model of the pad-disc braking system.

$A_{a}=\theta_{0}(R_{p}^{2}-r_{p}^{2})$ is the nominal contact area of the single pad with the disc, $R_{p}$, $r_{p}$ is the outer and inner radii of the brake pad, respectively, $2\theta_{0}$ is the cover angle of the pad and T(r,θ,z,t) is the spatial transient temperature field in the cylindrical coordinate system (r,θ,z) (Figure 1). Here and further, parameters and values corresponding with the pad and the disc are marked in “p” and “d”, respectively.

The solution to the initial value problem (5)–(8) takes the form:

(9) $$V(t)=V_{0}V^{*}(t),\;0\leq t\leq t_{s},$$

(10) $$V^{*}(t)=1-\frac{1}{t_{s}^{0}}\int_{0}^{t}F^{*}(\tau )d\tau$$

(11) $$t_{s}^{0}=\frac{mV_{0}}{8F_{0}}$$

The braking time $t_{s}$ is determined from the stopping condition $V(t_{s})=0$, which, taking into account formulas (9)–(11), gives the functional equation:

(12) $$\int_{0}^{t_{s}}F^{*}(t)dt=t_{s}^{0}$$

With the constant coefficient of friction $f=f_{0}$, $f^{*}(T)=1$ and immediate ($t_{i}\to 0$) pressure reaching the nominal value $p=p_{0}$, $p^{*}(t)=1$, we have $F^{*}(t)=1$ and from formulas (10) and (12) we find:

(13) $$V^{*}(t)=1-\frac{t}{t_{s}^{0}},\;0\leq t\leq t_{s}=t_{s}^{0}.$$

The next assumptions concern the formulation of the thermal problem of friction for a single disc brake, consisting of two identical pads each with the thickness $\delta_{p}$, pressed from two sides to the surfaces of the disc with internal $r_{d}$, external $R_{d}=R_{p}$ and thickness radii $2\delta_{d}$. Due to the symmetry of such a system relative to the center plane z=0 of the disc, to determine the temperature field it is enough to consider the friction system consisting of one pad sliding on the front surface of the disc of the thickness $\delta_{d}$ (Figure 1). As a result of friction, heat is generated in the pad contact area with the disc $\Gamma =\{r_{p}\leq r\leq R_{p},\;\left|\theta \right|\leq \theta_{0},\;z=0\}$ and the components heat up. The sum of the intensities of heat fluxes directed in the area of contact Γ from the friction surface along the normal inward the pad and disc is equal to the friction power density [26]:

(14) $$q(r,t)=f[T_{m}(t)]p(t)V(t)rR_{w}^{-1},\;r_{p}\leq r\leq R_{p},\;0\leq t\leq t_{s}.$$

We neglect the thermal resistance of the contact area, assuming even temperature of the friction surfaces of the pad and disc in that area. The surface of symmetry of the disc is adiabatic, and the free surfaces of the brake are cooled by convection with the constant heat transfer coefficient h averaged in the braking process [27,28]. The brake discs are solid and do not include an area for mounting on the wheel hub [29]. Such simplification is justified for short-term braking, when the temperature of the disc outside the friction path is insignificant.

With such assumptions, the transient temperature field T≡T(r,θ,z,t) can be found from the solution of the following spatial boundary value heat conduction problem (Figure 1):

(15) $$K_{p}\Delta T=\rho_{p}c_{p}\frac{\partial T}{\partial t},\;r_{p}<r<R_{p},\;\left|\theta \right|<\theta_{0},\;0<z<\delta_{p},\;0<t\leq t_{s},$$

(16) $$K_{d}\Delta T=\rho_{d}c_{d}\left[\frac{\partial T}{\partial t}+\frac{V(t)}{R_{w}}\frac{\partial T}{\partial \theta }\right],\;r_{d}<r<R_{d},\;\left|\theta \right|<\pi ,\;-\delta_{d}<z<0,\;0<t\leq t_{s},$$

(17) $${K_{d}\frac{\partial T}{\partial z}|}_{z=0^{-}}-{K_{p}\frac{\partial T}{\partial z}|}_{z=0^{+}}=q(r,t),\;(r,\;\theta )\in \Gamma ,\;0<t\leq t_{s},$$

(18) $$T(r,\theta ,0^{-},t)=T(r,\theta ,0^{+},t),\;(r,\;\theta )\in \Gamma ,\;0<t\leq t_{s},$$

(19) $${K_{p}\frac{\partial T}{\partial r}|}_{r=r_{p}}=h[T(r_{p},\theta ,z,t)-T_{0}],\;\left|\theta \right|<\theta_{0},\;0<z<\delta_{p},\;0<t\leq t_{s},$$

(20) $${K_{p}\frac{\partial T}{\partial r}|}_{r=R_{p}}=h[T_{0}-T(R_{p},\theta ,z,t)],\;\left|\theta \right|<\theta_{0},\;0<z<\delta_{p},\;0<t\leq t_{s},$$

(21) $${K_{p}\frac{1}{r}\frac{\partial T}{\partial \theta }|}_{\theta =-\theta_{0}}=h[T(r,-\theta_{0},z,t)-T_{0}],\;r_{p}<r<R_{p},\;0<z<\delta_{p},\;0<t\leq t_{s},$$

(22) $${K_{p}\frac{1}{r}\frac{\partial T}{\partial \theta }|}_{\theta =\theta_{0}}=h[T_{0}-T(r,\theta_{0},z,t)],\;r_{p}<r<R_{p},\;0<z<\delta_{p},\;0<t\leq t_{s},$$

(23) $${K_{p}\frac{\partial T}{\partial z}|}_{z=\delta_{p}}=h[T_{0}-T(r,\theta ,\delta_{p},t)],\;r_{p}<r<R_{p},\;\left|\theta \right|<\theta_{0},\;0<t\leq t_{s},$$

(24) $${K_{d}\frac{\partial T}{\partial r}|}_{r=r_{d}}=h[T(r_{d},\theta ,z,t)-T_{0}],\;\left|\theta \right|<\pi ,\;-\delta_{d}<z<0,\;0<t\leq t_{s},$$

(25) $${K_{d}\frac{\partial T}{\partial r}|}_{r=R_{d}}=h[T_{0}-T(R_{d},\theta ,z,t)],\;\left|\theta \right|<\pi ,\;-\delta_{d}<z<0,\;0<t\leq t_{s},$$

(26) $${K_{d}\frac{\partial T}{\partial z}|}_{z=0^{-}}=h[T_{0}-T(r,\theta ,0^{-},t)],\;r_{d}<r<r_{p},\;\left|\theta \right|<\pi ,\;0<t\leq t_{s},$$

(27) $${K_{d}\frac{\partial T}{\partial z}|}_{z=0^{-}}=h[T_{0}-T(r,\theta ,0^{-},t)],\;r_{p}<r<R_{p},\;\left|\theta \right|>\theta_{0},\;0<t\leq t_{s},$$

(28) $${\frac{\partial T}{\partial z}|}_{z=-\delta_{d}}=0,\;r_{d}<r<R_{d},\;\left|\theta \right|<\pi ,\;0<t\leq t_{s},$$

(29) $$T(r,\theta ,z,0)=T_{0},\;(r,\theta ,z)\in \Omega_{p}\cup \Omega_{d},$$

where q(r,t) is the friction power density (14) and Δ is the Laplace operator in the cylindrical coordinate system:

(30) $$\Delta \equiv \frac{\partial^{2}}{\partial r^{2}}+\frac{1}{r}\frac{\partial }{\partial r}+\frac{1}{r^{2}}\frac{\partial^{2}}{\partial \theta^{2}}+\frac{\partial^{2}}{\partial z^{2}}.$$

$\Omega_{p,d}$ is the spatial regions, occupied by the pad and the disc, respectively:

(31) $$\Omega_{p}=\{r_{p}\leq r\leq R_{p},\;\left|\theta \right|\leq \theta_{0},\;0\leq z\leq \delta_{p}\},\;\Omega_{d}=\{r_{d}\leq r\leq R_{d},\;\left|\theta \right|\leq \pi ,\;-\delta_{d}\leq z\leq 0\}.$$

$K_{p,d}$, $\rho_{p,d}$, $c_{p,d}$ are the thermal conductivities, densities and specific heat capacities of the pad and the disc materials, respectively.

Having the temperature field T(r,θ,z,t), the evolution of the bulk volumetric temperature of the pad $T_{V_{p}}$ and the disc $T_{V_{d}}$, we determine from the formulas:

(32) $$T_{V_{p}}(t)=\frac{1}{V_{p}}\int_{0}^{\delta_{p}}\int_{-\theta_{0}}^{\theta_{0}}\int_{r_{p}}^{R_{p}}T(r,\theta ,z,t)rdrd\theta dz,\;T_{V_{d}}(t)=\frac{1}{V_{d}}\int_{-\delta_{d}}^{0}\int_{-\pi }^{\pi }\int_{r_{d}}^{R_{d}}T(r,\theta ,z,t)rdrd\theta dz,$$

where $V_{p}=A_{a}\delta_{p}$, $V_{d}=\pi (R_{d}^{2}-r_{d}^{2})\delta_{d}$.

The change in thermomechanical wear of the friction surfaces during braking was calculated from the formula [30]:

(33) $$I_{w}(t)=A_{a}\int_{0}^{t}I[T_{m}(\tau )]f[T_{m}(\tau )]p(\tau )V(\tau )d\tau ,0\leq t\leq t_{s},$$

where f and I are the temperature-dependent $T_{m}$ (7) coefficients of friction and intensity of wear (1)–(3).

The above formulated problem of motion (8)–(12) and the thermal problem of friction (15)–(29) are coupled by the coefficient of friction $f[T_{m}(t)]$, which means that the sliding velocity V and temperature T are interdependent.

It should be also noted that the influence of microgeometry of the rubbing surfaces in determining the maximum temperature is related to the flash temperature, not only the mean temperature of the contact surfaces. It has been established that the flash temperature reaches the highest value in the initial period of braking and the mean surface temperature at about the midpoint of this period. The influence of topography of the surface on the temperature mode of a disc brake has been investigated in articles [15,31]. It was shown that the decisive influence on the maximum temperature has the mean temperature of the contact region of the pad with the disc. Hence, in the present study, the dependencies of the coefficient of friction and the wear rate only on the mean temperature of the contact region were used. There are also other approaches, which take into account the microgeometry of the rubbing surfaces. The roughness of these surfaces is usually simulated by introducing the thermal resistance into the boundary conditions and, as a result, by the appearance of temperature jump on the friction surface. In the proposed computational model, the solution of the boundary value problem of heat conduction was obtained under ideal (perfect) conditions of thermal contact of friction, which are typical for fairly smooth working surfaces of the pad and the disc. A review of the research on that topic is presented in article [32].

## 3. Numerical Analysis

The numerical solution of the system of equations of HDFW (1)–(32) was obtained using the finite element method adapted in COMSOL Multiphysics^®^ software [33]. To create the mesh of the brake, 8520 higher-order finite elements (quadratic Lagrange hexahedral elements) were used, including 1320 in the area occupied by the pad $\Omega_{p}$ and 7200 elements in the area of the brake disc $\Omega_{d}$ (Figure 1). The total number of degrees of freedom (DOF) of the model was equal to 78,307.

A computer simulation of frictional heating of the disc brake components during the single vehicle braking with the mass m=1524.3 kg from the initial velocity $V_{0}=100\;\mathrm{km}\;h^{-1}$ ($27.78\;m\;s^{-1}$) to standstill was performed. The pad and the disc dimensions were $R_{p}=R_{d}=113.5\;\mathrm{mm}$, $r_{p}=76.5\;\mathrm{mm}$, $r_{d}=66\;\mathrm{mm}$, $\delta_{d}=5.5\;\mathrm{mm}$, $\delta_{p}=10\;\mathrm{mm}$ and $\theta_{0}=32.25{}^{\circ}$ [34]. The value of the heat transfer coefficient was assumed to be equal $h=60\;W\;m^{-2}\;K^{-1}$, which is fully justified for short-term braking of a motor vehicle [28]. Initially, the disc and the pads were heated to the ambient temperature $T_{0}=20{}^{\circ}C$.

Calculations were made for the following variants, including the pads friction material and the nominal contact pressure [35]:

(1). 145-40: $p_{0}=0.588\;\mathrm{MPa}$;
(2). 42-773: $p_{0}=0.588\;\mathrm{MPa}$;
(3). 2-61: $p_{0}=0.588\;\mathrm{MPa}$;
(4). FMC-11: (a) $p_{0}=0.588\;\mathrm{MPa}$; (b) $p_{0}=1.47\;\mathrm{MPa}$;
(5). MCV-50: (a) $p_{0}=0.49\;\mathrm{MPa}$; (b) $p_{0}=1.47\;\mathrm{MPa}$;
(6). FC-16L: (a) $p_{0}=0.392\;\mathrm{MPa}$; (b) $p_{0}=1.47\;\mathrm{MPa}$.

The characteristics of the material of the pads, necessary to carry out calculations, are given in Table 1. For the material of the brake disc, ChNMKh gray cast iron ($K_{d}=52.17\;W\;m^{-1}\;K^{-1}$, $\rho_{d}=7100\;\mathrm{kg}\;m^{-3}$, $c_{d}=444.6\;J\;{\mathrm{kg}}^{-1}\;K^{-1}$) was chosen.

From the selected brake pad materials, two groups can be distinguished, the first consisting of four materials and marked with numbers 1, 2, 3 and 6 (145-40, 42-773, 2-61 and FC-16L). They are characterized by low thermal conductivity ($K_{p}=0.39÷0.79\;W\;m^{-1}\;K^{-1}$), the lowest density ($\rho_{p}=2300÷2500\;\mathrm{kg}\;m^{-3}$) and the highest specific heat capacity ($c_{p}=961÷1206\;J\;{\mathrm{kg}}^{-1}\;K^{-1}$). Materials 145-40, 42-773 and 2-61 represent a combined binder formed from mixtures of the same name and differing in proportions of ingredients such as asbestos, synthetic resin, rubber, barite, aluminum oxide, graphite, copper powder and brown shavings. Retinax FC-16L type A (variant 6) is a composite based on phenol-formaldehyde resins and reinforced with brass shavings.

The second group contains two sintered cermet friction materials FMC-11 (64% Fe, 15% Cu, 3% SiO_2_, 6% BaSO_4_, 3% asbestos and 9% graphite) and MCV-50 (64% Fe, 10% Cu, 5% B_4_C, 5% SiC, 5% FeSO_4_, 3% asbestos), belonging to variants 4 and 5, respectively. The distinctive for them are significantly higher than in the first group, the values of the thermal conductivity (35 $W\;m^{-1}\;K^{-1}$ and 30.78 $W\;m^{-1}\;K^{-1}$, respectively), density is almost twice as high (≈5000) and there is lower specific heat capacity, on average $c_{p}\approx 478\;J\;{\mathrm{kg}}^{-1}K^{-1}$.

It is necessary to emphasize that modern friction materials are complex multi-component systems. Their composition and manufacturing technology are usually not disclosed. In the scientific literature only data on the averaged (effective) thermophysical and mechanical properties of the materials as a whole or their individual components were available. The methods for determining such averaged characteristics are known but are not the subject of investigations in the present study. The composite materials used in this thermal analysis are very different, and it would be difficult to take into account the inhomogeneity of the properties of each of them.

The results of calculations shown below in figures for each of the six abovementioned variants have been marked with numbers 1, 2, …, 6, respectively. In addition, the curves corresponding to the calculation variants 4, 5 and 6 at low nominal pressure values ($p_{0}=0.588\;\mathrm{MPa}$, 0.49 MPa, 0.392 MPa) were denoted as 4a, 5a or 6a (solid lines). In contrast, the curves obtained for the same variants at high nominal pressure ($p_{0}=1.47\;\mathrm{MPa}$) were denoted as 4b, 5b or 6b (dashed lines).

**Figure 2:**
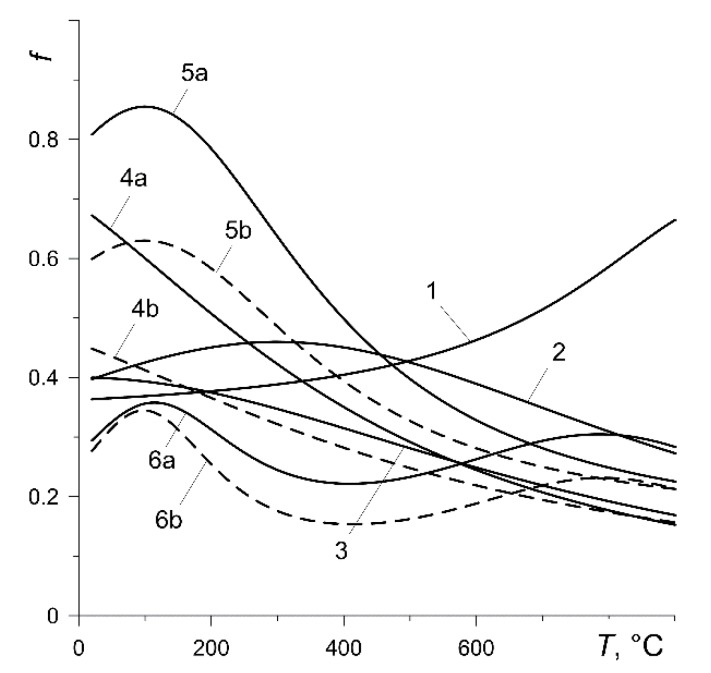
Experimental curves of thermal friction stability for six calculation variants [35].

**Figure 3:**
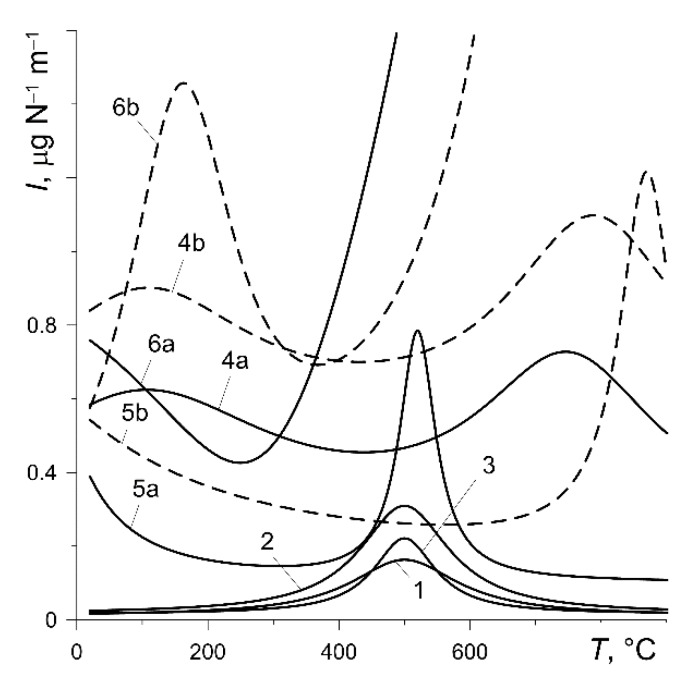
Experimental dependence of thermomechanical wear intensity *I* on temperature *T* for six calculation variants [35].

The values of the coefficients of friction $f_{0}$ and intensity of wear $I_{0}$ at the initial temperature $T_{0}=20{}^{\circ}C$ and the values of the coefficients $f_{i}$, $I_{i}$, i=1,2,…,7, in formulas (2) and (3), approximating the experimental data, are given in Table 2 [31,35]. The experimental dependencies (1)–(3) of the coefficient of friction f and the intensity of wear I on the temperature T for all calculation variants are presented in Figure 2 and Figure 3, respectively. The friction thermostability curves of selected materials differ significantly (Figure 2). Increase in temperature causes a monotonic increase in the coefficient of friction in the case of the pads made of material 145-40 (variant 1) or its almost linear decrease for the pads made of materials 2-61 (variant 3) and FMC-11 (variant 4). Friction thermostability curves for pads made of 42-773 (variant 2) and MCV-50 (variant 5) have a local maximum and, with Retinax FC-16L (variant 6), a local minimum. At the set temperature, the coefficient of friction is greater at lower contact pressure (variants 4, 5 and 6).

According to formula (33), the value of the wear intensity factor I has a decisive impact on the material wear of a particular friction pair during braking. Experimental dependence I(T) for the six pad materials considered are shown in Figure 3. Except for the pad made of Retinax FC-16L (variant 6), the values of I do not exceed $1.2\;\mu g\;N^{-1}\;m^{-1}$ in the temperature range of 20 ÷ 900 °C. At a temperature not exceeding 400 °C, the friction pairs of variants 1, 2 and 3 are characterized by a very low (below $0.14\;\mu g\;N^{-1}\;m^{-1}$) wear intensity. A rapid increase in wear intensity after reaching the temperature of 400 °C is shown by the friction pair with the pad made of FC-16L (variant 6). At a temperature of around 500 °C, the wear intensity shows a clear maximum for variants 1, 2, 3, 5a and 5b. At the specified temperature, the wear intensity is higher at the higher contact pressure (variants 4, 5 and 6).

**Figure 4:**
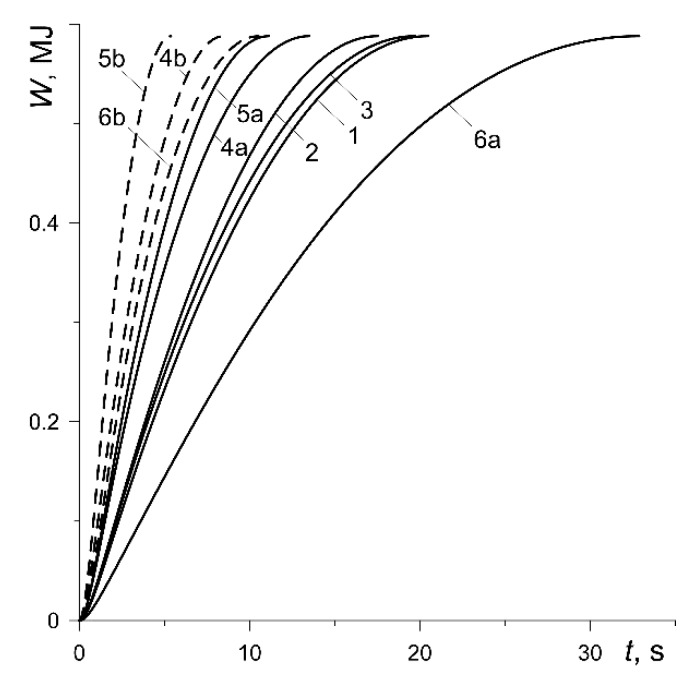
Evolutions of the work done W during braking.

The evolution of the work done by each out of the four braking systems of the motor vehicle:

(34) $$W(t)=8A_{a}\int_{0}^{t}q(r_{eq},\tau )d\tau ,0\leq t\leq t_{s},$$

where equivalent radius $r_{eq}$ and friction power density q were obtained from formula (8) and (14), respectively and are shown in Figure 4. It increases monotonically from zero to the nominal value $W_{0}\equiv W(t_{s})=0.588\;\mathrm{MJ}$, the same for each considered calculation variant. The fastest (5.38 s) value of $W_{0}$ is achieved in the case of the friction pair with the MCV-50 pad at nominal pressure $p_{0}=1.47\;\mathrm{MPa}$ (curve 5b), and the slowest (32.88 s) was when the pad material was Retinax FC-16L at the nominal pressure $p_{0}=0.392\;\mathrm{MPa}$ (curve 6a).

**Figure 5:**
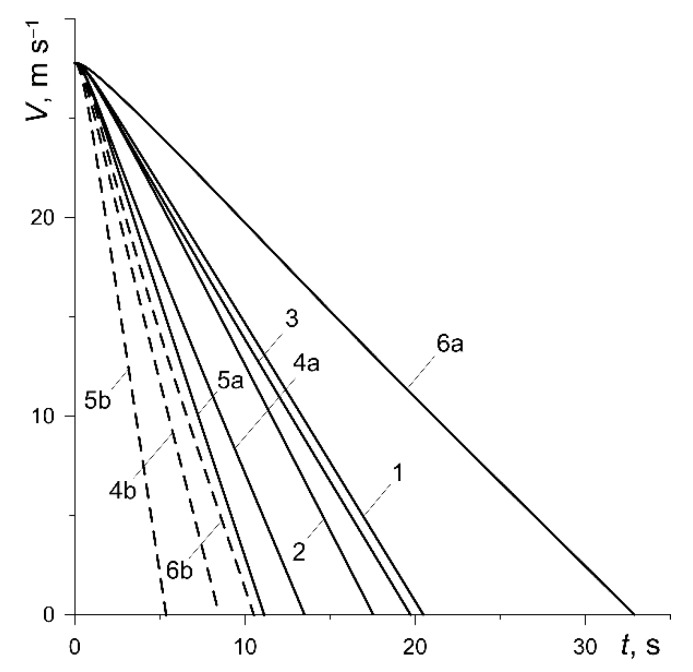
Changes in the vehicle velocity V during braking.

The reduction in vehicle velocity from the initial value $V_{0}=27.78\;m\;s^{-1}$ to zero at the time of stopping for all variants is shown in Figure 5. Except for the short initial period, the reduction in velocity is linear, which was previously also established for thermally sensitive materials in article [31]. The longest ($t_{s}=32.88\;s$) braking lasted in the case of the friction pair from variant 6, and the shortest ($t_{s}=5.38\;s$) was from variant 5 at low pressure $p_{0}=0.49\;\mathrm{MPa}$ (Table 3). Increasing the contact pressure to the value $p_{0}=1.47\;\mathrm{MPa}$ significantly reduces the braking time: 37.1% (variant 4), 51.7% (variant 5), 68% (variant 6).

**Figure 6:**
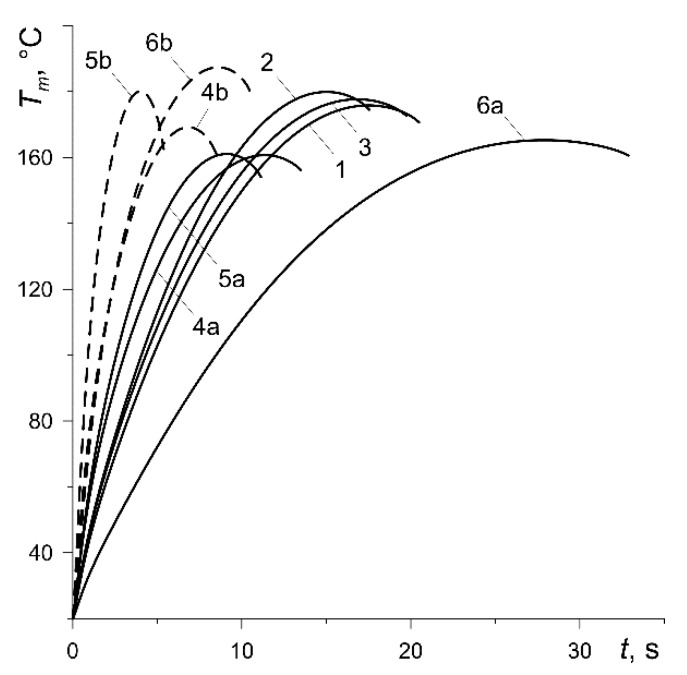
Evolutions of the average temperature $T_{m}$ of the contact area of the pad with the disc.

Evolutions of the mean temperature $T_{m}$ (7) of the contact area of the pad with the disc are presented in Figure 6. Due to the same initial kinetic energy and, therefore, the total work done (Figure 4), the maximum values $T_{m,\max}$ differ slightly for all considered calculation variants. The difference between the highest 187.4 °C (curve 6b) and the lowest 160.6 °C (curve 4b) is equal to about 14.3% (Table 3).

**Figure 7:**
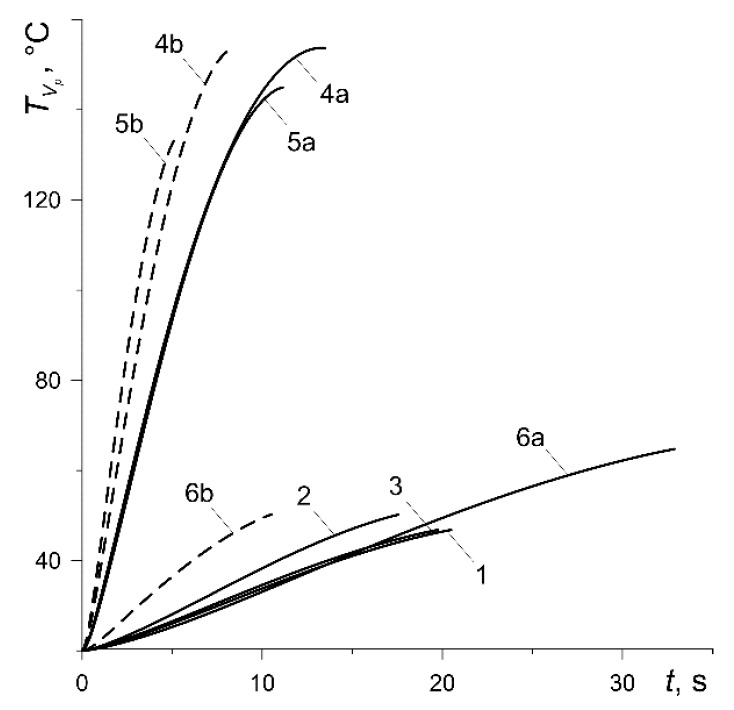
Time profiles of the bulk volumetric temperature $T_{V_{p}}$ of the pad.

**Figure 8:**
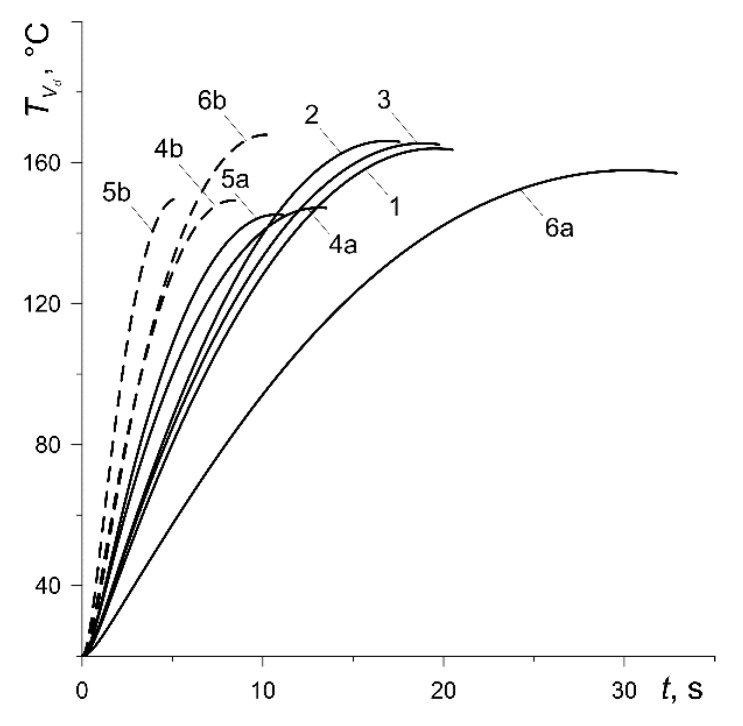
Time profiles of the bulk volumetric temperature $T_{V_{d}}$ of the disc.

Changes in the bulk volumetric temperature of the pad $T_{V_{p}}$ and the disc $T_{V_{d}}$ (32) with the braking time are shown in Figure 7 and Figure 8, respectively. The maximum values of the temperature for the pad made of materials with low thermal conductivity (variants 1, 2, 3, 6) are low and fluctuate from 47 °C (curve 3) to 65 °C (curve 6a) (Figure 7). The values of the thermal conductivity for cermet FMC-11 (variant 4) and MCV-50 (variant 5) are much higher, which means that the maximum values of their bulk temperature are higher and vary in the range from 134 °C (curve 5b) to 154 °C (curve 4a). The maximum values of the bulk volumetric disc temperature are equalized for all calculation variants and vary from 145 °C (variant 5a) to about 168 °C (variant 6b) (Figure 8). It should be noted that the results presented in Figure 7 and Figure 8 are very much related to the values of the heat partition coefficient, which in turn are determined by the thermophysical properties of friction pair materials [36]. With a significantly lower thermal conductivity of the pad, more heat is directed from the friction surface to the disc, and hence, the volumetric temperature of the disc is higher than the pad temperature. Due to the similar thermophysical properties of the pad materials with the disc material (variants 4, 5), their bulk temperatures differ slightly.

However, an insignificant difference between the mean temperature of the friction surface (Figure 6) and bulk temperature of the pad (Figure 7) takes place only in the case of ceramic metal pads (variants 4 and 5). This is explained by their greater (by two orders of magnitude) thermal conductivity compared with the other four materials (variants 1–3, 6). Consequently, the effective heating depth of the ceramic metal pads is also substantially greater than the rest of the materials (they heat up over the entire thickness). High thermal conductivity of the cast-iron disc and almost twice smaller computational thickness than the pad are the reasons for the small difference in the corresponding bulk and surface temperatures shown in Figure 6 and Figure 8.

We note that evolution of the temperature of the friction surface of the disc has an oscillating nature [20,22,31]. This is caused by the motion of the contact region along the working surface of the disc due to its rotation. At each revolution of the disc, the temporal profile of the temperature of the specified point on the friction surface of the disc consists of two stages—increasing and decreasing. First, when the point of the rubbing path of the disc comes into contact with the pad, the temperature increases and reaches the maximum value, then after passing through the pad area, it decreases until another contact with the pad takes place. In the present analysis, the purpose was to find the change in the mean temperature of the contact region with respect to braking time defined by formula (7). That temperature was used in the construction of the computational model. Since it is associated with the stationary pad, there are no oscillations. The other reason is the averaging of temperature within the contact region, and therefore, we can see only the monotonic change in the mean (and volumetric) temperatures in Figure 6, Figure 7 and Figure 8.

**Figure 9:**
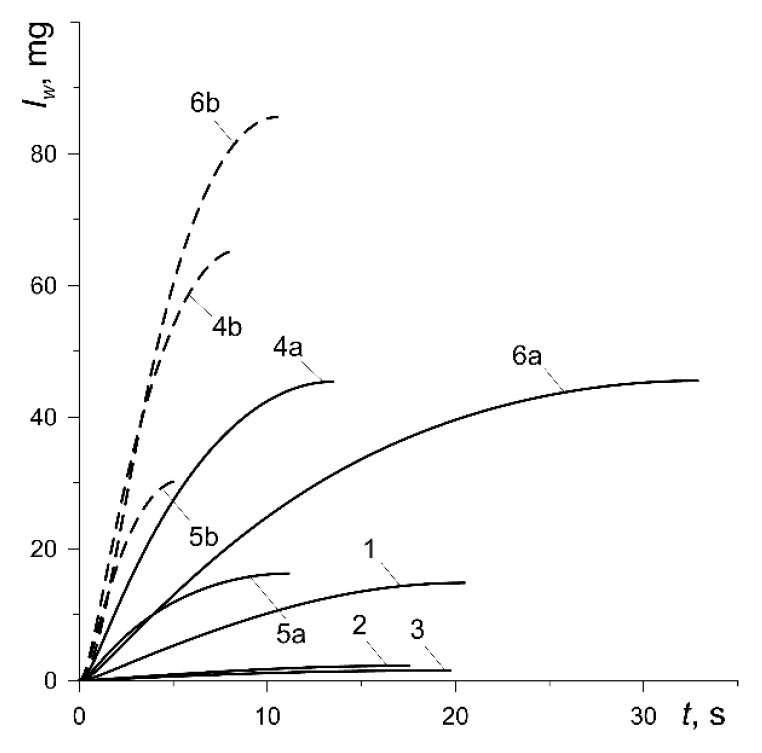
Evolutions of the thermomechanical mass wear $I_{w}$ during braking.

The thermomechanical mass wear of the friction surfaces $I_{w}$ (33) increases monotonically from zero at the initial time moment to the maximum value at the stopping (Figure 9). The lowest total wear in the process takes place for the friction pairs including pads made of materials 42-773 (curve 2, $I_{w,\max}=2.22\;\mathrm{mg}$) and 2-61 (curve 3, $I_{w,\max}=1.51\;\mathrm{mg}$), and the largest when using the pads made of Retinax FC-16L (curve 6b, $I_{w,\max}=85.56\;\mathrm{mg}$) (Table 3). Increasing the contact pressure (variants 4, 5, 6) causes significant (even several times) increase in wear.

**Figure 10:**
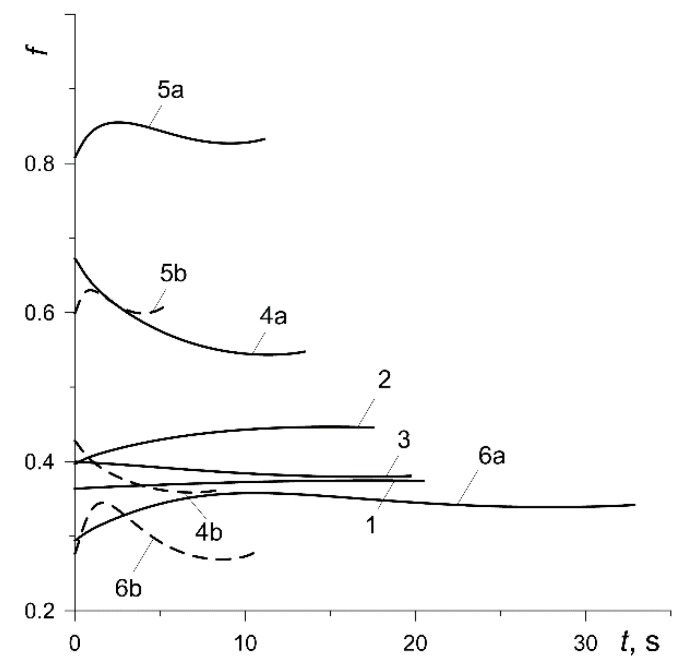
Time profiles of the coefficient of friction f.

Knowing the time profiles of the mean temperature $T_{m}$ (7) of the six analyzed calculation variants (Figure 6), the formulas for the coefficient of friction f (Figure 10) were reproduced using formulas (1) and (2). Relatively low (below 200 °C) maximum values of $T_{m}$, presented in Table 3, cause the initial values of $f=f_{0}$ to be approximately maintained throughout the entire braking process. The coefficient of friction for the pad made of 145-40 (variant 1) was stable, a slight increase in f occurred for the friction pair from variant 2, and a decrease occurred for variant 3. The largest changes in the coefficient of friction during a single braking were observed in the results obtained from calculations according to materials denoted 4, 5 and 6.

**Figure 11:**
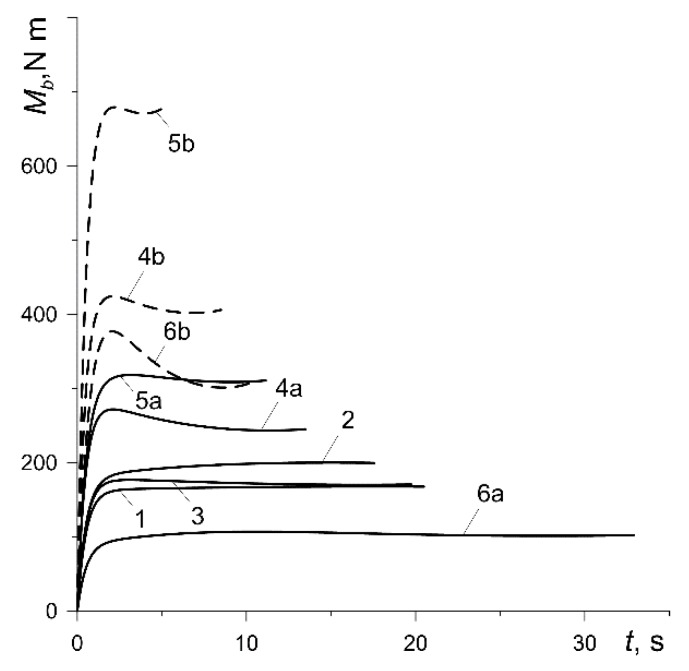
Time courses of the braking torque $M_{b}$.

The time profiles of the braking torque from one vehicle braking system $M_{b}(t)=2F(t)r_{eq}$, where the friction force F(t) and the equivalent radius of the contact surface of the disc $r_{eq}$ were determined from formulas (6) and (8), respectively, are similar to the changes in the coefficient of friction presented in Figure 10 (Figure 11). Just as the friction force, the braking torque, depends on the contact pressure p(t) (4) and therefore increases from zero to a certain constant value within about 2 s. At lower nominal pressure values $p_{0}$ (curves 1, 2, 3, 4a, 5a, 6a), $M_{b}$ remains stable practically at the same level throughout the entire process. However, at high pressure $p_{0}=1.47\;\mathrm{MPa}$ (curves 4b, 5b and 6b), the time profiles of the braking torque after reaching the maximum value reveal a decrease with time to the minimum and then a slight increase again until the vehicle stops. That effect is the most noticeable in the case of the pad made of Retinax FC-16L (curve 6b).

The results obtained from the computer simulations are summarized in Table 3 and Table 4. They contain the parameters that determine the operation of the selected friction pair of the braking system: braking time $t_{s}$, maximum values of the mean temperature of the contact area $T_{m,\max}$, bulk volumetric temperature of the pad $T_{V_{p},\max}$ and disc $T_{V_{d},\max}$, thermomechanical mass wear $I_{w,\max}$ (Table 3), mean value of the coefficient of friction $f_{m}$, its stability $f_{s}$ and fluctuation $f_{f}$, as well as braking efficiency $\alpha_{eff}$ (Table 4).

The temperature field calculated numerically allows the conclusion that the temperature mode (regime) of the brake is light [36]. It occurs when the bulk volumetric temperature of the friction pair is about 100 °C and the average temperature of the friction surface does not exceed 200 °C. Materials for which the temperature of the beginning of destruction of at least one of the components is in the range 250÷300 °C are suitable for operation in such conditions. Thus, the thermal stability of separate components has a significant impact on the frictional stability of the material, determining the permissible temperature modes of its operation. It should also be noted that with light temperature modes, the wear of the disc is usually not taken into account and the service life of the braking system and reliability are mainly determined by its temperature mode.

Based on the comparative analysis of the data contained in Table 3 and Table 4, it is apparent that among the pad materials belonging to the first of the abovementioned groups (variants 1, 2, 3 and 6), friction material 42-773 (variant 2) is the best presented. When using that material for the pads in combination with the cast-iron brake disc, the braking time is the shortest, the maximum temperature of the friction surface slightly differs from the values obtained for the other materials in that group, the mass wear is low whereas the average value of the coefficient of friction $f_{m}$, stability of the coefficient of friction ($f_{s}$) and its fluctuation ($f_{f}$) are the highest (required) during the braking process. The friction pair from variant 2 also has the highest braking rate in that group. Among the friction materials from the second group (variants 4 and 5), the optimum choice with both low and high contact pressure is cermet MCV-50 (variant 5). It excels in the absolute ranking of all six friction pairs, ahead of the cermet FMC-11 in the second place. It should be noted that a significant disadvantage of friction ceramic metal materials is their high hardness and rigidity. Hence, as shown also in the results in Table 3, the wear of the pad material and its bulk volumetric temperature increase significantly and their noise characteristics deteriorate.

It should be noted that the present study examines the so-called light temperature mode, when the temperature of the friction surface does not exceed 200 °C. This is caused by the properties of the materials of the pads employed in the calculations. The first three (variants 1–3) can be used only with light frictional heating modes. The ceramic metal materials (variants 4 and 5), as well as Retinax (variant 6), can withstand much higher temperatures such as medium (about 450 °C) and heavy (above 750 °C) temperature modes. This research was limited only to the light mode for all six materials, because the main objective was to perform a comparative analysis of the obtained characteristics of these materials operating under the same conditions (the same initial velocity, pressure and full work done). However, the proposed method of initial selection of the pad material remains unchanged in the rest of the temperature modes. It should also be noted thatthere is too little information about the experimental data for the temperature dependence of the coefficients of friction and the intensity of wear. Such data was found and used for six pad materials in combination with the same cast-iron disc.

## 4. Conclusions

The aim of the present study was to develop a methodology for the initial material selection for brake pads based on temperature mode data. Numerical simulation of the friction heating process of the disc brake during single braking was carried out. The basis for calculations was the coupled spatial system of equations of HDFW taking into account the thermal sensitivity of the coefficients of friction and the intensity of thermomechanical mass wear. The numerical solution using FEM of the developed system of equations of HDFW was obtained for six selected pad materials (six calculation variants), combined with the cast-iron brake disc while maintaining a constant work done during braking. As a result, the key parameters of the braking process were determined, such as the braking time and time profiles of the mean temperature of the nominal contact area of the pad with the disc and their bulk volumetric temperature, thermomechanical mass wear, coefficient of friction and braking torque. This allowed the maximum values of the mean and bulk temperatures as well as mass wear for six selected pads materials to be determined. Four parameters were considered for the pads material selection with the best friction characteristics, taking into account the change in the coefficient of friction: average value, stability, fluctuation and braking efficiency. Based on the initial material selection, carried out by means of comparative analysis of the values of these parameters for each of the six friction pairs, it was found that the best tribological characteristics have MCV-50, FMC-11 ceramic metal materials and material denoted as 42-773. The final choice of the pads material should also be based on the strength tests, technology, production costs of the material and the ecological aspects.

We proposed the general method of initial selection of the pads material for the given material of the disc. The calculations were made for the light temperature mode of the brake operation and were aimed only to illustrate this method. The presented technique is quite universal, and calculations on it can be carried out for medium and heavy temperature modes too, including the use of complicated mathematical models taking into account the topography of the surface of friction, the temperature dependence of the properties of materials, nonuniformity of contact pressure distribution, etc.

## Figures and Tables

**Table 1:** Thermophysical properties of materials of the pads [35].

No.	Pads Material	$K_{p},\;W\;m^{-1}\;K^{-1}$	$\rho_{p},\;\mathrm{kg}\;m^{-3}$	$c_{p},\;J\;{\mathrm{kg}}^{-1}\;K^{-1}$
1	145-40	0.49	2500	1206
2	42-773	0.51	2300	961
3	2-61	0.39	2500	961
4	FMC-11	35	4700	478.9
5	MCV-50	30.78	5300	511.6
6	FC-16L	0.79	2500	961

**Table 2:** Coefficients in the approximation formulas (1)–(3) [31,35].

Pads Material	$p_{0},\;\mathrm{MPa}$	$f_{0}$	$f_{1}$	$f_{2}$	$f_{3}$	$f_{4}$	$f_{5}$	$f_{6}$	$f_{7}$
		$I_{0}$	$I_{1}$	$I_{2}$	$I_{3}$	$I_{4}$	$I_{5}$	$I_{6}$	$I_{7}$
145-40	0.588	0.364	0.915	1.016	$3.4\times 10^{-3}$	1000	0	0	0
		0.017	0.626	8.997	$1\times 10^{-2}$	500	0	0	0
42-773	0.588	0.397	0.105	1.053	$1.5\times 10^{-3}$	300	0	0	0
		0.025	0.656	11.75	$1.2\times 10^{-2}$	500	0	0	0
2-61	0.588	0.4	0	1	$1.3\times 10^{-3}$	0	0	0	0
		0.019	0.84	10.85	$1.75\times 10^{-2}$	500	0	0	0
FMC-11	0.588	0.672	$9.45\times 10^{-3}$	1.134	$1.9\times 10^{-3}$	–180	0	0	0
		0.584	0.377	0.644	$4\times 10^{-3}$	100	0.786	$6\times 10^{-3}$	750
	1.471	0.45	$8.04\cdot 10^{-2}$	1.071	$1.5\times 10^{-3}$	−250	0	0	0
		0.839	0.602	0.437	$5\times 10^{-3}$	105	0.672	$6.2\times 10^{-3}$	790
MCV-50	0.49	0.808	0.167	0.891	$3.3\times 10^{-3}$	100	0	0	0
		0.39	0.251	3.138	$1.5\times 10^{-2}$	−100	1.726	$3\times 10^{-2}$	520
	1.471	0.45	0.08	0.26	$-2.16\times 10^{-3}$	−167	0.73	$1.756\times 10^{-3}$	−106
		0.543	0.282	1.877	$0.4\times 10^{-2}$	−300	1.877	$2\times 10^{-2}$	870
FC-16L	0.392	0.294	0	0.973	$5.5\times 10^{-3}$	105	0.973	$2.5\times 10^{-3}$	800
		0.759	1.306	7.736	$6\times 10^{-3}$	800	−1.451	$4\times 10^{-3}$	300
	1.471	0.28	0.072	1.041	$7\times 10^{-3}$	95	0.723	$3\times 10^{-3}$	800
		1.479	0	3.412	$0.8\times 10^{-2}$	850	0.93	$0.4\times 10^{-2}$	0

**Table 3:** Characteristics of the temperature mode and temperature wear of the friction pairs.

No.	Pads Material	$t_{s}\;[s]$		$T_{m,\max}\;[{}^{\circ}C]$		$I_{w,\max}\;[\mathrm{mg}]$		$T_{V_{p},\max}\;[{}^{\circ}C]$		$T_{V_{d},\max}\;[{}^{\circ}C]$	
	Contact Pressure: Low (a), High (b)	a	b	a	b	a	b	a	b	a	b
	Required Value	Low		Low		Low		Low		Low	
1	145-40	20.49	–	175.7	–	14.81	–	46.8	–	164	–
2	42-773	17.54	–	179.8	–	2.22	–	50.2	–	166.2	–
3	2-61	19.73	–	177.7	–	1.51	–	46.8	–	165.5	–
4	FMC-11	13.5	8.49	160.5	169.1	45.37	65.34	153.7	153.7	147.2	149.2
5	MCV-50	11.14	5.38	161.1	180.1	16.23	30.35	144.9	134.7	145.2	149.7
6	FC-16L	32.88	10.53	165.3	187.4	45.52	85.56	64.7	50.3	157.8	167.9

**Table 4:** Braking performance parameters.

No.	Pads Material	$f_{m}$		$f_{s}=f_{m}/f_{\max}$		$f_{f}=f_{\min}/f_{\max}$		$\alpha_{eff}=f_{s}/t_{s}^{2}\;[s^{-2}]$	
	Contact Pressure: Low (*a*), High (*b*)	a	b	a	b	a	b	a	b
	Required Value	High		High		High		High	
1	145-40	0.372	–	0.991	–	0.970	–	0.0024	–
2	42-773	0.435	–	0.973	–	0.889	–	0.0032	–
3	2-61	0.387	–	0.967	–	0.950	–	0.0025	–
4	FMC-11	0.575	0.374	0.855	0.875	0.808	0.838	0.0047	0.0121
5	MCV-50	0.838	0.611	0.980	0.970	0.945	0.951	0.0079	0.0335
6	FC-16L	0.343	0.297	0.960	0.861	0.822	0.780	0.0009	0.0078

## Nomenclature

$A_{a}$	surface area of the nominal contact region [$m^{2}$]
c	specific heat capacity [$J\;{\mathrm{kg}}^{-1}\;K^{-1}$]
f	coefficient of friction
$f_{f}$	fluctuation of the coefficient of friction
$f_{s}$	stability of the coefficient of friction
h	convective heat transfer coefficient [$W\;m^{-2}\;K^{-1}$]
I	temperature-dependent coefficient of wear intensity [$\mu {\mathrm{gN}}^{-1}m^{-1}$]
$I_{w}$	thermomechanical mass wear [mg]
K	thermal conductivity [$W\;m^{-1}\;K^{-1}$]
m	vehicle mass [kg]
p	contact pressure [MPa]
$p_{0}$	nominal contact pressure [MPa]
q	heat flux density [$W\;m^{-2}$]
r	radial coordinate
r,R	inner and outer radius, respectively [m]
$r_{eq}$	equivalent radius of the contact region [m]
$R_{w}$	dynamic rolling radius of the wheel [m]
t	time [s]
$t_{i}$	rise time [s]
$t_{s}$	braking time [s]
T	temperature [°C]
$T_{0}$	initial temperature [°C ]
$T_{a}$	ambient temperature [°C ]
$T_{m}$	mean temperature on the nominal contact region [°C ]
V	vehicle velocity [$m\;s^{-1}$]
$V_{0}$	initial vehicle velocity [$m\;s^{-1}$]
W	work done during braking [J]
z	axial coordinate
**Greek symbols**	
$\alpha_{eff}$	braking efficiency [$s^{-2}$]
$\beta_{eff}$	relative braking efficiency [$s^{-2}m^{-1}$]
δ	thickness of the analyzed region [m]
θ	circumferential coordinate
$\theta_{0}$	cover angle of the pad [deg]
ρ	density [$\mathrm{kg}\;m^{-3}$]
ω	angular velocity of the disc [$\mathrm{rad}\;s^{-1}$]
$\omega_{0}$	initial angular velocity of the disc [$\mathrm{rad}\;s^{-1}$]
**Subscripts**	
d	denotes disc
p	denotes pad

## References

[1] Maleque M.A., Dyuti S., Rahman M.M. Material selection method in design of automotive brake disc. Proceedings of the World Congress on Engineering.

[2] Shanian A., Milani A.S., Carson C., Abeyaratne R.C. (2008). A new application of ELECTRE III and revised Simos’ procedure for group material selection under weighting uncertainty. Knowl. Based Syst..

[3] Dieter G.E. (2000). Engineering Design.

[4] Farag M.M. (2008). Materials and Process Selection for Engineering Design.

[5] Ashby M.F. (2005). Materials Selection in Mechanical Design.

[6] Mohammadnejad A., Bahrami A., Goli M., Dehbashi Nia H., Taheri P. (2019). Wear induced failure of automotive disc brakes—A case study. Materials.

[7] Sayeed Ahmed G.M., Algarni S. (2018). Design, Development and FE thermal analysis of a radially grooved brake disc developed through direct metal laser sintering. Materials.

[8] Newcomb T.P., Spurr R.T. (1967). Braking of Road Vehicles.

[9] Ścieszka S.F. (1998). Fricion Brakes.

[10] Parafiniak M. (2014). Investigation and development of friction materials dedicated to high-energy loaded aircraft wheel brakes. Logistyka Nauka.

[11] Chichinadze A.V. (2009). Theoretical and practical problems of thermal dynamics and simulation of the friction and wear of tribocouples. J. Frict. Wear.

[12] Day A.J. (2014). Braking of Road Vehicles.

[13] Chichinadze A.V., Braun E.D., Ginzburg A.G., Ignat’eva E.V. (1979). Calculation, Testing and Selection of Friction Couples.

[14] Chichinadze A.V. (1995). Processes in heat dynamics and modelling of friction and wear (dry and boundary friction). Tribol. Int..

[15] Grzes P. (2017). Determination of the maximum temperature at single braking from the FE solution of heat dynamics of friction and wear system of equations. Numer. Heat Transf. Part A.

[16] Chichinadze A.V., Kozhemjakina V.D., Suvorov A.V., Kokonin S.S. (2009). Application of theories of thermal dynamics and modeling of a friction and wear process of firm bodies at designing of the hard loaded brakes of transport cars. Frict. Lubr. Mach. Mech..

[17] Chichinadze A.V., Okulov B.S., Suvorov A.V., Sverchkov J.H., Bakin A.I., Mozalev V.V. (2001). The basic principles of optimal testing of heavy-loaded brakes of transport vehicles. Russ. J. Heavy Mach..

[18] Yevtushenko A.A., Grzes P. (2012). Axisymmetric finite element model for the calculation of temperature at braking for thermosensitive materials of a pad and a disc. Numer. Heat Transf. Part A.

[19] Yevtushenko A.A., Grzes P. (2012). Axisymmetric FEA of temperature in a pad/disc brake system at temperature-dependent coefficients of friction and wear. Int. Commun. Heat Mass Transf..

[20] Yevtushenko A.A., Adamowicz A., Grzes P. (2013). Three-dimensional FE model for the calculation of temperature of a disc brake at temperature-dependent coefficients of friction. Int. Commun. Heat Mass Transf..

[21] Yevtushenko A.A., Grzes P. (2015). Effect of dimensions of pad and disk on the temperature and duration of braking. J. Frict. Wear.

[22] Yevtushenko A.A., Grzes P. (2015). 3D FE model of frictional heating and wear with a mutual influence of the sliding velocity and temperature in a disc brake. Int. Commun. Heat Mass Transf..

[23] Trzepiecinski T., Lemu H.G. (2019). A three-dimensional elastic-plastic contact analysis of Vickers indenter on a deep drawing quality steel sheet. Materials.

[24] Bermudo C., Sevilla L., Castillo López G. (2017). Material flow analysis in indentation by two-dimensional digital image correlation and finite elements method. Materials.

[25] Ginzburg A.G. (1973). Theoretical and experimental bases of calculation of single process of braking by means of system of the equations of thermal dynamics of friction. Optimal Use of Friction Materials in Friction Couples of Machines.

[26] Ling F.F. (1973). Surface Mechanics.

[27] Adamowicz A., Grzes P. (2011). Influence of convective cooling on a disc brake temperature distribution during repetitive braking. Appl. Therm. Eng..

[28] Adamowicz A., Grzes P. (2012). Convective cooling of a disc brake during single braking. Acta Mech. Autom..

[29] Talati F., Jalalifar S. (2009). Analysis of heat conduction in a disk brake system. Heat Mass Transf..

[30] Ginzburg A.G., Chichinadze A.V. (1977). To calculation of wear during braking using the equations of thermal dynamics of friction. Friction and Wear of Friction Materials.

[31] Grzes P. (2018). Finite element solution of the three-dimensional system of equations of heat dynamics of friction and wear during single braking. Adv. Mech. Eng..

[32] Yevtushenko A.A., Kuciej M. (2012). One-dimensional thermal problem of friction during braking: The history of development and actual state. Int. J. Heat Mass Transf..

[33] COMSOL Multiphysics^®^ v. 5.3. www.comsol.com. www.comsol.com.

[34] Adamowicz A. (2016). Effect of convective cooling on temperature and thermal stresses in disk during repeated intermittent braking. J. Frict. Wear.

[35] Chichinadze A.V., Matveevskii R.M., Braun E.P. (1986). Materials in Triboengineering of Unsteady Processes.

[36] Balakin V.A., Sergienko V.P. (1999). Heat Calculations of Brakes and Friction Units.

